# Spontaneous Heterotopic Pregnancy: A Case Report of a Potentially Life-Threatening Condition

**DOI:** 10.7759/cureus.67488

**Published:** 2024-08-22

**Authors:** Vladimir Valencia, Rachel M Worcester, Afrah S Abedi, Emma Majewski, Vy Pham, Natasha Eliz

**Affiliations:** 1 School of Medicine, Lake Erie College of Osteopathic Medicine, Bradenton, USA; 2 Obstetrics and Gynecology, St. Vincent's Medical Center, Jacksonville, USA; 3 Obstetrics and Gynecology, Lake Erie College of Osteopathic Medicine, Bradenton, USA

**Keywords:** ectopic pregnancy, coincident pregnancy, multiple‑sited pregnancy, combined ectopic pregnancy, heterotopic pregnancy

## Abstract

Heterotopic pregnancy involves the coexistence of both an intrauterine and an extrauterine pregnancy occurring simultaneously. The spontaneous incidence of heterotopic pregnancy in the general population has been estimated to be 1/30,000. This report discusses the case of a 37-year-old female who presented to the emergency department with vaginal bleeding and lower abdominal pain. Upon workup, a transabdominal and transvaginal ultrasound demonstrated a heterotopic pregnancy with an estimated gestational age of seven weeks. The ultrasounds confirmed an intrauterine pregnancy with fetal cardiac activity and a fetal pole along with a right adnexal ectopic pregnancy with fetal cardiac activity and a fetal pole. Additionally, a right paratubal cyst was incidentally found. The patient subsequently underwent exploratory laparotomy with a right salpingectomy for the removal of the right tubal pregnancy and a right paratubal cyst. This case denotes the significance of a carefully performed ultrasound examination, especially in the early weeks of pregnancy. Even when a standard pregnancy is visualized with ultrasound examination, it remains imperative for the examiner to meticulously examine the adnexa and interstitial portion of the fallopian tube. A heterotopic pregnancy has the potential to be life-threatening and can often go undetected, resulting in missed diagnoses.

## Introduction

Heterotopic pregnancy involves the coexistence of both an intrauterine and an extrauterine pregnancy occurring simultaneously. Extrauterine pregnancy is most commonly found in the fallopian tube but may also be seen in the cervix or ovary. The spontaneous incidence of heterotopic pregnancy in the general population has been estimated to be 1/30,000 [1]. The incidence of heterotopic pregnancy has been shown to have increased in women trying to conceive with assisted reproduction technology, such as in vitro fertilization (IVF), and is estimated to be up to 1/3900. Additional risk factors in the general population are the same as those for ectopic pregnancy and often include a history of pelvic inflammatory disease (PID), prior abdominal or pelvic surgery, Müllerian abnormality of the uterus, use of an intrauterine device, smoking, and in utero diethylstilbestrol exposure [2].

Patients with heterotopic pregnancy typically present with a triad of pelvic pain, vaginal bleeding, and amenorrhea, but can be asymptomatic or non-specific [3]. Pelvic or generalized abdominal pain is the most frequent symptom due to peritoneal irritation, but vaginal bleeding may not always present in clinical presentation [4]. In more serious cases, a patient may present with shock due to a rupture of the ectopic pregnancy. A clinical presentation of a heterotopic pregnancy can closely imitate the clinical symptoms of a threatened abortion or isolated ectopic pregnancy, which makes early detection and diagnosis of such cases very difficult. Because these symptoms occur in a variety of non-gynecological and gynecological conditions, it is required to rule out more detrimental causes.

Heterotopic pregnancy is usually not detected until the patient is symptomatic or is further along in gestational age. Measurement of beta-human chorionic gonadotropin (β-hCG) concentrations often provides a diagnostic clue that a heterotopic pregnancy is taking place. A β-hCG level that is irregularly high for the intrauterine estimated fetal age warrants further investigation. Ultrasound examination is a key diagnostic tool to assess for both intrauterine gestation and extrauterine pregnancy. However, transvaginal ultrasound is only about 56% sensitive at 5-6 weeks gestation [5]. For heterotopic pregnancies of less than six weeks gestation, a diagnosis may be made with the presence of an ectopic fetal heartbeat alone.

## Case presentation

The patient was a healthy 37-year-old G3P1011 (gravida 3, term 1, preterm 0, abortion 1, living 1) female with no known significant prior past medical history. She admitted to a prior vaginal delivery and denied prior surgical history; however, a prior Pfannenstiel abdominal scar was noted on examination. Of note, the patient’s primary language was Portuguese, and a virtual medical translator was present to facilitate the care of the patient. She presented to the emergency department with complaints of sharp lower abdominal pain and vaginal bleeding. Symptom onset was four days prior, and she described passing small amounts of dark blood during the episodes. She had no prior history of these symptoms. The patient denied fever, chills, nausea, or vomiting. At the time, she believed herself to be four weeks pregnant. This pregnancy occurred spontaneously without prior fertility treatment.

Initial vital signs were stable, with a blood pressure of 111/67 mmHg, heart rate of 98 beats per minute (bpm), respiratory rate of 19 breaths per minute, and an oxygen saturation of 100%. The physical exam was unremarkable.

Laboratory values were overall unremarkable and are listed in Table 1.

**Table 1:** Laboratory values

Lab Values	Value	Reference Range (1st Trimester)
Endocrinology		
Beta-Human Chorionic Gonadotropin (β-hCG)	142,946 mIU/mL	32,000 - 210,000 mIU/mL
Complete Blood Count / Differential		
White Blood Cell Count	7 x 10³/μL	5.7 - 13.6 x 10³/μL
Red Blood Cell Count	3.97 10⁶/μL	3.42 - 4.55 x 10⁶/μL
Hemoglobin	12.4 g/dL	11.6 - 13.9 g/dL
Hematocrit	37.70%	31.00 - 41.00%
Mean Corpuscular Volume (MCV)	95 μm³	81 - 96 μm³
Mean Corpuscular Hemoglobin (MCHC)	32.9% Hb/cell	31 - 36% Hb/cell
Red Cell Distribution Width (RDW)	13.5%	12.5 - 14.1%
Platelet Count	256 x 10⁹/L	150 - 400 x 10⁹/L
Mean Platelet Volume (MPV)	7.2 μm³	7.7 - 10.3 μm³
Neutrophils - Absolute	4.5 x 10³/mm³	3.6 -10.1 x 10³/mm³
Lymphocytes - Absolute	1.9 x 10³/mm³	1.1 - 3.6 x 10³/mm³
Monocytes - Absolute	0.3 x 10³/mm³	0.1 - 1.1 x 10³/mm³
Eosinophils - Absolute	0.1 x 10³/mm³	0.0 - 0.6 x 10³/mm³
Basophils - Absolute	0 x 10³/mm³	0.0 - 0.1 x 10³/mm³
Routine Chemistry		
Sodium	137 mEq/L	133 - 148 mEq/L
Potassium	3.9 mEq/L	3.6 - 5.0 mEq/L
Chloride	106 mEq/L	101 - 105 mEq/L
Blood Urea Nitrogen	7 mg/dL	7 - 12 mg/dL
Creatinine	0.6 mg/dL	0.4 - 0.7 mg/dL
Anion Gap	9 mmol/L	13 - 17 mmol/L
Osmolality	285 mOsm/kg H₂O	275 - 280 mOsm/kg H₂O
Calcium	10.0 mg/dL	8.8 - 10.6 mg/dL
Estimated Glomerular Filtration Rate (eGFR)	118 mL/min	131 - 166 mL/min

**Figure 1:**
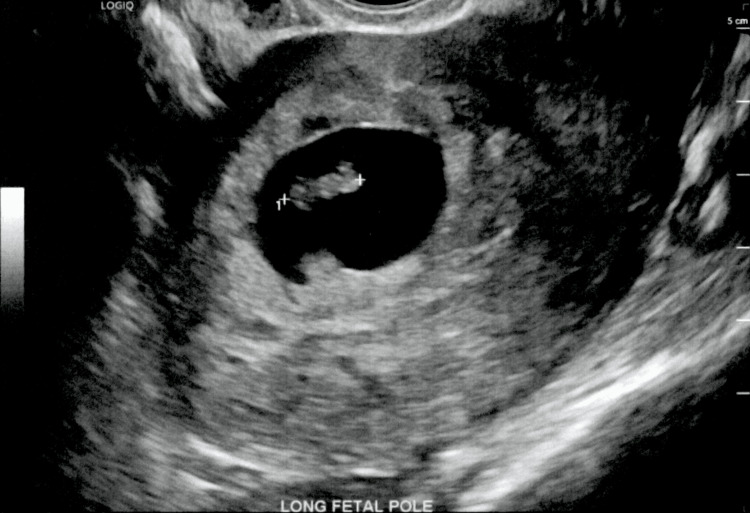
TVUS of the long fetal pole showing an intrauterine gestation with the crown-rump length measuring 9.8 mm TVUS: transvaginal ultrasound

A transabdominal and transvaginal grayscale, color Doppler, and M-mode sonography of the pelvis were performed (Figure 1). Findings showed a single intrauterine gestation present with the crown-rump length measuring 9.8 mm, which corresponded to a gestational age of 7 weeks and 0 days. M-mode imaging demonstrated a fetal heart rate of 132 bpm. A subchorionic hemorrhage was also noted measuring 1.7 x 0.9 x 1.3 cm.

**Figure 2:**
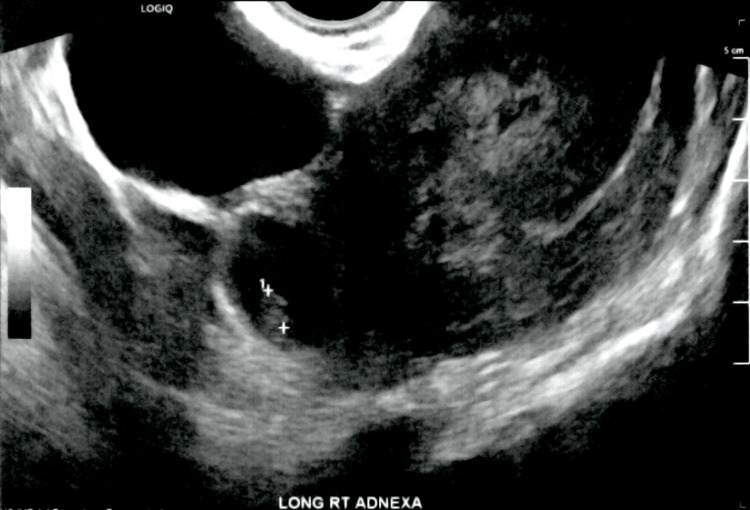
TVUS of the long right adnexa with an ectopic pregnancy measuring 2.3 cm in diameter with crown-rump length measuring 6.9 mm TVUS: transvaginal ultrasound

In the right adnexal region (Figure 2), a complex cystic structure containing a yolk sac and fetal pole consistent with an ectopic pregnancy measuring 2.3 cm in diameter was noted. The crown-rump length measured 6.9 mm corresponding to a gestational age of 6 weeks and 4 days. M-mode imaging demonstrated a fetal heart rate of 112 bpm. No complex ascites was identified related to the pregnancy. Initially, a right ovarian cyst measuring 7.2 cm was believed to be present; however, this was later found to be a simple paratubal cyst.

**Figure 3:**
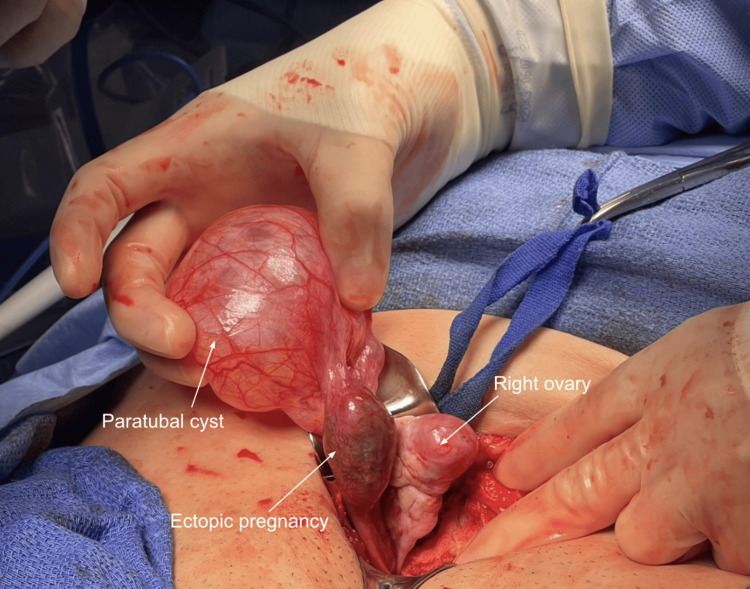
Visualization of the paratubal cyst, ectopic pregnancy, and right ovary

After extensive discussion about the patient’s therapeutic options, she consented to an exploratory laparotomy, which demonstrated the paratubal cyst along with the ectopic pregnancy (Figure 3). The patient subsequently underwent right salpingectomy and removal of right tubal pregnancy and right paratubal cyst on day 0 of admission. Surgery was uncomplicated with successful removal of the ectopic pregnancy in the ampullary region of the tube along with removal of the large 7 cm paratubal cyst. The estimated blood loss was 30 mL, and there were no complications overall with the procedure. 

The patient responded well to the surgery and was admitted for overnight observation. The ectopic pregnancy in the right adnexa, along with the paratubal cyst were sent off to pathology. Pathology confirmed the presence of an ectopic pregnancy and a simple paratubal cyst. The patient was discharged on day 1 of admission in a stable fashion.

## Discussion

Spontaneous heterotopic pregnancy, the concurrent development of intrauterine and extrauterine gestations, remains a rare but clinically significant condition [3]. Its incidence has increased, particularly with the widespread use of assisted reproductive technologies (ART) and ovarian stimulation for infertility treatment, along with other risk factors such as pelvic inflammatory disease (PID), pelvic surgery, and previous fallopian tube damage [3]. The case presented highlights the rare occurrence of spontaneous heterotopic pregnancy despite harboring any known risk factors. There are several complexities and challenges associated with diagnosing and managing this condition, which, if left untreated, can lead to severe maternal complications and mortality [6].

A systematic review conducted by Talbot et al. reported that 33% of heterotrophic cases had prior imaging of a normal intrauterine pregnancy, which led to false reassurance and misdiagnoses [1]. Cerniauskaite et al. reported that 70% of all heterotopic pregnancies are diagnosed between 5-8 weeks of gestation, 20% between 9-10 weeks, and 10% past the 11th week, making it vital to detect as early as possible. The early diagnosis of heterotopic pregnancy is challenging because an elevated β-hCG level and an intrauterine embryo seen on ultrasound point to a successful intrauterine pregnancy, leading to no concern of ectopic pregnancy. Therefore, it is essential to identify both adnexa to ensure no existence of extrauterine gestation. If no conclusive adnexal findings are seen on ultrasound, other diagnostic features, including free fluid in the peritoneum or pelvis, hematosalpinx, and hemoperitoneum, would aid in the diagnosis of an ectopic pregnancy. The identification of ectopic pregnancy on TVUS has a reported sensitivity of 71-100% and a specificity of 41-99% [7]. Studies reported a wide interval for β-hCG concentrations for the diagnosis and management of heterotopic pregnancies. Values of β-hCG can range from 105,000 to 106,520 mIU/mL in both heterotopic and normal intrauterine pregnancies at around six weeks gestation [8]. Therefore, the presence of a markedly elevated β-hCG, as in this patient at 142,946 mIU/mL, should clue the observer to search for a coexistent pregnancy. Higher than expected β-hCG requires persistent monitoring with repeat β-hCG levels and ultrasounds to check for multiple gestations or heterotopic pregnancy.

Additionally, being thorough with ultrasound examinations is crucial. This is to ensure the detection of heterotopic pregnancies in the presence of a paratubal cyst, which may otherwise go unnoticed. A meticulous approach involves careful examination of the pelvic region, utilizing various ultrasound techniques such as transabdominal and transvaginal imaging. Attention to detail in assessing both the ovaries and the uterus is essential, as the presence of a paratubal cyst can obscure the visualization of ectopic pregnancy [9]. Familiarity with ultrasound appearances of different types of cysts and ectopic pregnancies, coupled with a systematic scanning protocol, enhances the likelihood of early detection and appropriate management. Regular training, staying updated with the latest guidelines, and maintaining a high level of vigilance during ultrasound examinations are essential components of ensuring comprehensive patient care and preventing diagnostic oversights.

The mainstay treatment of heterotopic pregnancies requires consideration of the patient's hemodynamic stability, prognosis or desired outcome, and site of implantation of ectopic pregnancy [8]. If the patient is hemodynamically stable, laparoscopy is preferred over laparotomy as it is less invasive. The first line of treatment is salpingectomy to remove the extrauterine pregnancy, but it is vital to ensure proper methods to preserve the existing intrauterine pregnancy. If the intrauterine pregnancy is desired and the patient is hemodynamically stable, the patient can undergo transvaginal injection of feticidal substances, such as potassium chloride or hyperosmolar glucose, which are often tolerated by the intrauterine pregnancy [10]. This method has been documented in cases in which heterotopic pregnancy was recognized early in gestation, reiterating the importance of early detection of heterotopic pregnancy.

## Conclusions

Spontaneous heterotopic pregnancy, though rare, is a potentially life-threatening condition that requires a high degree of clinical suspicion for timely diagnosis and treatment. This case emphasizes the importance of considering heterotopic pregnancy in patients with abdominal pain and vaginal bleeding, even without traditional risk factors. Accurate diagnosis through transvaginal ultrasound and serial serum β-hCG measurements is crucial to prevent severe maternal complications. Surgical intervention is typically necessary to resolve the ectopic pregnancy while preserving the intrauterine pregnancy. Continued education and research are vital to improving the recognition, management, and prognosis of spontaneous heterotopic pregnancy, along with implementing the most optimal strategy to improve maternal and fetal outcomes.

## References

[1] Talbot K, Simpson R, Price N, Jackson SR (2011). Heterotopic pregnancy. J Obstet Gynaecol.

[2] Soares C, Maçães A, Novais Veiga M, Osório M (2020). Early diagnosis of spontaneous heterotopic pregnancy successfully treated with laparoscopic surgery. BMJ Case Rep.

[3] Oancea M, Ciortea R, Diculescu D (2020). Spontaneous heterotopic pregnancy with unaffected intrauterine pregnancy: systematic review of clinical outcomes. Medicina (Kaunas).

[4] Dündar O, Tütüncü L, Müngen E, Muhcu M, Yergök YZ (2006). Heterotopic pregnancy: tubal ectopic pregnancy and monochorionic monoamniotic twin pregnancy: a case report. Perinatal Journal.

[5] Ankum WM, Van der Veen F, Hamerlynck JV, Lammes FB (1993). Transvaginal sonography and human chorionic gonadotrophin measurements in suspected ectopic pregnancy: a detailed analysis of a diagnostic approach. Hum Reprod.

[6] Elsayed S, Farah N, Anglim M (2023). Heterotopic pregnancy: case series and review of diagnosis and management. Case Rep Obstet Gynecol.

[7] Seffah JD (2000). Ultrasonography and ectopic pregnancy — a review. Int J Gynaecol Obstet.

[8] Nguyen KP, Hudspeth M, Milestone H (2022). Spontaneous heterotopic pregnancy: diagnosis and management. Case Rep Obstet Gynecol.

[9] Teka H, Yemane A, Gebremeskel M, Kinfe BA, Kiros S, Kidanu M (2023). Heterotopic pregnancy with ipsilateral adnexal cyst causing a diagnostic dilemma: a case report. Int Med Case Rep J.

[10] Allison JL, Aubuchon M, Leasure JD, Schust DJ (2012). Hyperosmolar glucose injection for the treatment of heterotopic ovarian pregnancy. Obstet Gynecol.

